# Outcomes of Patients with Cirrhosis Undergoing Cardiac Defibrillator Placement: A Nationwide Analysis

**DOI:** 10.7759/cureus.80614

**Published:** 2025-03-15

**Authors:** Ahmed Younes, Hossam Albeyoumi, Ahmed K Mahmoud, Ibrahim Kamel, Ahmed M Maraey, Mahmoud Khalil

**Affiliations:** 1 Internal Medicine, Riverside Shore Memorial Hospital, Onancock, USA; 2 Internal Medicine, University of Connecticut School of Medicine, New Haven, USA; 3 Internal Medicine, Boston University School of Medicine, Boston, USA; 4 Internal Medicine, Tufts University School of Medicine and Tufts Medical Center, Carney Hospital, Boston, USA; 5 Cardiology, Toledo University, Toledo, USA; 6 Cardiology, University of Connecticut School of Medicine, New Haven, USA

**Keywords:** database hcup nis nrd research, implantable cardiac defibrillator (icd), liver cirrhosis, patient-centered outcomes research, patient outcomes

## Abstract

Background

Invasive procedures pose a greater risk for patients with liver cirrhosis. This study investigates the impact of cirrhosis on the outcomes of implantable cardiac defibrillator (ICD) implantation.

Methods

We conducted a retrospective analysis using the National Readmissions Database (NRD) from 2016 to 2020. Adult patients who received an ICD, identified by the International Statistical Classification of Diseases and Related Health Problems 10^th^ Revision (ICD-10) codes, were included. Outcomes were compared between patients with and without underlying liver cirrhosis. The primary outcome was all-cause inpatient mortality. Secondary outcomes included ischemic cerebrovascular accidents (CVA), major bleeding (gastrointestinal, intracranial, pulmonary, and other bleeding), packed red blood cell (pRBC) transfusion, pericardial complications (pericardial effusion, hemopericardium, or pericardial tamponade), acute kidney injury (AKI), acute myocardial infarction (AMI), length of stay, and total hospital charges.

Results

Among 264,518 patients who underwent defibrillator implantation, 3,507 patients (1.3%) had liver cirrhosis. Patients with cirrhosis experienced significantly higher inpatient mortality (adjusted odds ratio (aOR): 2.29, 95% confidence interval (CI): 1.70-3.08, P<0.001), major bleeding (aOR: 2.40, 95% CI: 1.97-2.91, P<0.001), pRBC transfusion (aOR: 2.19, 95% CI: 1.81-2.64, P<0.001), pericardial complications (aOR: 1.37, 95% CI: 1.05-1.79, P=0.02), and AKI (aOR: 1.44, 95% CI: 1.29-1.59, P<0.001). No significant difference was observed in the incidence of ischemic CVA (aOR: 0.89, 95% CI: 0.33-2.43, p=0.83), but there was a reduced incidence of AMI (aOR: 0.69, 95% CI: 0.59-0.84, P<0.001) in patients with cirrhosis. Additionally, liver cirrhosis was associated with increased hospital stays (adjusted mean difference (aMD): 2.79 days, 95% CI: 2.20-3.37, P<0.001) and higher total charges (aMD: $35,624, 95% CI: 23,698-47,549, P<0.001).

Conclusion

Cirrhosis is associated with increased mortality, bleeding complications, and greater resource utilization after ICD implantation. These results emphasize the need for careful evaluation when considering this procedure in patients with liver cirrhosis.

## Introduction

Implantable cardioverter defibrillators (ICDs) are a cornerstone therapy for preventing sudden cardiac death in high-risk patients. Survival benefits have been demonstrated in clinical trials and real-world clinical practice data [1].

Cirrhosis is a significant global public health issue with an increasing prevalence. Recent estimates indicate that between 107 and 119 million people worldwide have cirrhosis, with about 2.4% of global deaths related to it [2,3]. Patients with cirrhosis often experience systemic complications that present considerable clinical challenges. Among these is cirrhotic cardiomyopathy, which increases the risk of arrhythmias and heart failure [4].

Patients with liver cirrhosis undergoing invasive procedures generally face increased risks of adverse outcomes, particularly those with higher Child-Pugh classes or Model for End-Stage Liver Disease (MELD) scores exceeding 13 [5]. However, there is limited data on the safety and outcomes of ICD implantation in this population. 

Utilizing a large national database in this study, we aim to evaluate the risk of patients with cirrhosis undergoing ICD placement to better inform clinical decision-making.

## Materials and methods

Data sources, study population, and outcomes

This retrospective study utilizes the National Readmission Database (NRD) from 2016 to 2020 [6]. The NRD is a comprehensive, public, all-payer database provided by the Agency for Healthcare Research and Quality (AHRQ) that includes more than 50% of hospitalizations in the United States [7]. To ensure the generalizability of results, discharge weights were applied to each record.

The study included adult patients (aged >18 years) who underwent ICD placement during the study period. Patients were identified using the appropriate International Statistical Classification of Diseases and Related Health Problems 10^th^ Revision (ICD-10) codes [8] detailed in Appendix A. Outcomes were then compared between patients with and without liver cirrhosis.

Key patient characteristics examined included age, sex, hypertension, diabetes mellitus, smoking, peripheral vascular disease (PVD), chronic kidney disease (CKD), heart failure, history of coronary artery disease (CAD) (defined as a history of myocardial infarction, percutaneous coronary intervention, or coronary artery bypass graft), atrial fibrillation (AF), primary insurance type, and median income for the patient’s Zone Improvement Plan (ZIP) code. Hospital-level characteristics included bed size, location, and teaching status. Baseline characteristics are summarized in Table 1.

The primary outcome was all-cause inpatient mortality, while secondary outcomes included ischemic cerebrovascular accidents (CVA), major bleeding (defined as gastrointestinal, intracranial, pulmonary, and other bleeding), packed red blood cell (pRBC) transfusion, pericardial complications (defined as pericardial effusion, hemopericardium, or pericardial tamponade), acute kidney injury (AKI), length of stay (LOS), and total charges.

Statistical analysis

In this study, categorical variables were expressed as frequencies and percentages, with comparisons performed using chi-square tests. Continuous variables were expressed as means and standard deviations, and comparisons were conducted using Student's t-tests. Logistic regression was employed to analyze dichotomous and categorical variables, while linear regression was used for continuous variables. Multivariate models were developed for both logistic and linear regressions.

The models were adjusted for age, sex, hypertension, diabetes mellitus, smoking, PVD, CKD, heart failure, history of CAD, AF, and primary insurance type. These variables were selected based on their statistical significance (P<0.05) in univariate analyses of baseline characteristics against the primary outcome. A P-value threshold of <0.05 was considered statistically significant for all analyses. This study did not require ethical approval as no data from human subjects or animals were collected.

## Results

**Figure 1:**
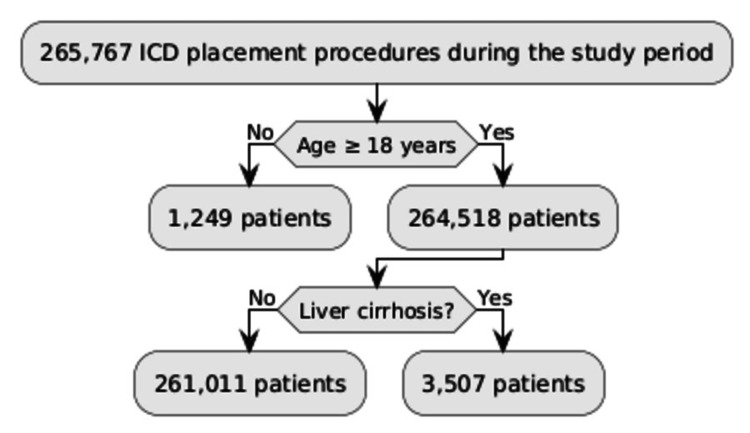
Flowchart summarizing patient population ICD: implantable cardiac defibrillator

Among 264,518 patients who underwent ICD placement, 3,507 patients (1.3%) had liver cirrhosis. Baseline characteristics are summarized in Table 1. The flow chart summarizing the patient population is in Figure 1.

**Table 1:** Baseline characteristics of the study group ICD: implantable cardiac defibrillator; PVD: peripheral vascular disease; CKD: chronic kidney disease; CAD: coronary artery disease Continuous variables were compared using Student's t-tests, while dichotomous and categorical variables were compared using logistic regression.

	ICD with no cirrhosis N=261,011 (98.7%)	ICD with cirrhosis N = 3,507 (1.3%)	P-value
Age, mean, and SD	65.7 (±0.2)	62.8 (±0.5)	<0.001
Female (%)	74,314 (28.4%)	735 (20.9%)	<0.001
Primary insurance (%)			<0.001
Medicare	160,662 (61.6)	2,098 (59.9)	
Medicaid	28,757 (11)	700 (19.9)	
Private insurance	57,003 (21.9)	515 (14.7)	
Self-pay	6,055 (2.3)	80 (2.3)	
No charge/other	8,212(3.1)	110 (3.1)	
Median household income for patient's zip code (%)			<0.001
0-25^th^ percentile	77,090 (30.0)	1,243 (35.9)	
26^th^-50^th^ percentile	70,548 (27.4)	877 (25.4)	
51^st^-75^th^ percentile	61,274 (23.8)	810 (23.4)	
76^th^-100^th^ percentile	48,472 (18.8)	527 (15.3)	
Hospital bed size (%)			0.03
Small	23,334 (8.9)	249 (7.1)	
Medium	65,282 (25)	892 (25.4)	
Large	172,396 (66.1)	2,367 (67.5)	
Hospital location and teaching status (%)			0.28
Metropolitan non-teaching	42,175 (16.1)	617 (17.6)	
Metropolitan teaching	212,345 (81.4)	2,809 (80.1)	
Non-metropolitan hospital	6,492 (2.5)	81 (2.3)	
Hypertension (%)	53,648 (20.6)	435 (12.4)	<0.001
Diabetes mellitus (%)	42,339 (16.2)	825 (23.5)	<0.001
Tobacco smoking (%)	38,107 (14.6)	729 (20.8)	<0.001
PVD (%)	17,572 (6.7)	272 (7.8)	0.09
CKD (%)	76,964 (29.5)	1,364 (38.9)	<0.001
Heart failure (%)	224,828 (86.1)	3,212 (91.6)	<0.001
History of CAD (%)	107,838 (41.3)	1,264 (36)	<0.001
Atrial fibrillation (%)	109,295 (41.9)	1,734 (49.4)	<0.001

Primary outcomes

Liver cirrhosis was associated with significantly increased inpatient mortality (adjusted odds ratio (aOR): 2.29, 95% confidence interval (CI): 1.70-3.08, P<0.001).

Secondary outcomes

**Figure 2:**
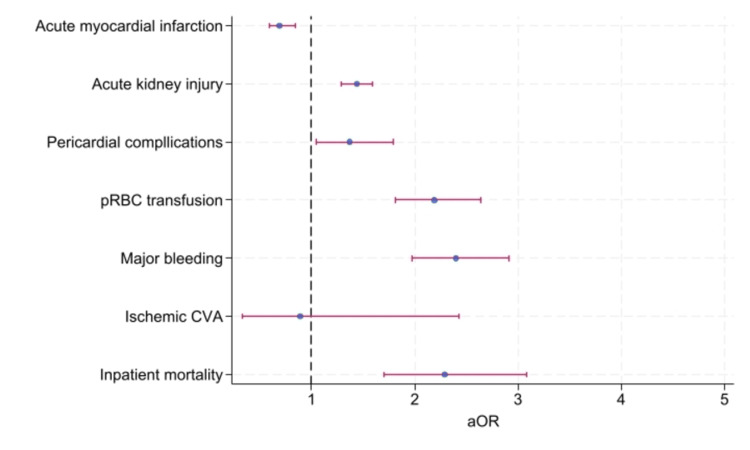
Forest plot of the primary and secondary outcomes pRBC: packed red blood cell; CVA: cerebrovascular accident; aOR: adjusted odds ratio

Liver cirrhosis was associated with significantly increased major bleeding (aOR: 2.40, CI: 1.97-2.91, P<0.001), pRBC transfusion (aOR: 2.19, CI: 1.81-2.64, P<0.001), pericardial complications (aOR: 1.37, CI: 1.05-1.79, P=0.02), and AKI (aOR: 1.44, CI: 1.29-1.59, P<0.001), without significant difference in ischemic CVA (aOR: 0.89, CI: 0.33-2.43, p=0.83) and with reduced incidence of AMI (aOR: 0.69, CI: 0.59-0.84, P<0.001). The primary and secondary outcomes are summarized in Table 2 and the forest plot is in Figure 2.

**Table 2:** Primary and secondary outcomes ICD: implantable cardiac defibrillator; CVA: cerebrovascular accident; pRBC: packed red blood cell; LOS: length of stay; SD: standard deviation Continuous outcomes were compared using linear regression, while dichotomous outcomes were compared using logistic regression.

Parameters	ICD without cirrhosis N=261,011 (98.7%)	ICD with cirrhosis N = 3,507 (1.3%)	aOR/aMD (95% CI)	p-value
Inpatient mortality (%)	2,876 (1.1%)	94 (2.7%)	2.29 (1.70 - 3.08)	<0.001
Ischemic CVA (%)	484 (0.2%)	6 (0.2%)	0.89 (0.33 - 2.43)	0.83
Major bleeding (%)	6,424 (2.5%)	222 (6.3%)	2.40 (1.97-2.91)	<0.001
pRBC transfusion (%)	8,609 (3.3%)	260 (7.4%)	2.19 (1.81 - 2.64)	<0.001
Pericardial complications (%)	5,579 (2.1)	101 (2.9)	1.37 (1.05 - 1.79)	0.02
Acute kidney injury (%)	78,186 (29.9)	1,494 (42.6)	1.44 (1.29 - 1.59)	<0.001
Acute myocardial infarction (%)	30,042 (11.5)	287 (8.2)	0.69 (0.59 - 0.84)	<0.001
LOS, days, mean (SD)	8.3 (±0.1)	11.9 (±0.6)	2.79 (2.20 - 3.37)	<0.001
Total charges, $, mean (SD)	241,723 (±4,923)	287,661 (±13,596)	35,624 (23,698 - 47,549)	<0.001

Resource utilization

Liver cirrhosis was also associated with increased resource utilization with increased LOS (adjusted mean difference (aMD): 2.79 days, CI: 2.20-3.37, P<0.001), and total charges (aMD: $35,624, CI: 23,698-47,549, P<0.001).

## Discussion

This study utilized the NRD from 2016 to 2020 [6] to evaluate the impact of liver cirrhosis on outcomes after ICD placement. We found that patients with cirrhosis are generally at increased risk of inpatient mortality and postprocedural complications.

Implantable cardiac defibrillators have demonstrated a survival benefit, reducing the risk of sudden cardiac death in high-risk patients [1]. However, our study shows that liver cirrhosis is associated with about a two-fold increased risk of inpatient mortality after ICD placement. This finding aligns with the understanding that cirrhosis is associated with multiple systemic complications, including cirrhotic cardiomyopathy, which can increase the risk of arrhythmias and heart failure [4]. Furthermore, patients with advanced liver disease, particularly those with higher Child-Pugh classes or MELD scores exceeding 13, generally face increased risks of adverse outcomes after invasive procedures [5].

We also found that liver cirrhosis is associated with a significant increase in major bleeding and the need for pRBC transfusion. This is consistent with the known coagulopathy and altered hemostasis commonly observed in patients with cirrhosis [9]. Impaired hepatic synthetic function, portal hypertension, reduced platelet count and function, and increased fibrinolysis can contribute to an increased risk of bleeding in this population [10].

Our study identified a higher risk of pericardial complications, including pericardial effusion and tamponade, in cirrhotic patients. While pericardial complications are rare in the general ICD population, cirrhotic patients may be more susceptible due to homeostatic imbalances, venous congestion, and a propensity for pericardial effusion associated with advanced liver disease [11]. Previous studies have reported an increased risk of pericardial effusion following cardiovascular interventions in cirrhotic patients, further supporting our findings [12]. 

Patients with cirrhosis face a significantly elevated risk of developing AKI due to systemic complications associated with their liver disease. The hallmark hyperdynamic circulation in cirrhosis leads to reduced effective blood volume, triggering vasoconstrictive responses and chronic renal hypoperfusion [13]. This makes cirrhotic patients more vulnerable to AKI from common events like infections or bleeding [14,15]. Factors such as nephrotoxic medications, intrinsic kidney diseases, and cholemic nephropathy further compound the risk of AKI in this population [16].

Interestingly, our study found that cirrhosis is associated with a decreased risk of AMI. This finding is consistent with previous research suggesting that cirrhotic patients have a lower prevalence of AMI despite their high burden of cardiovascular risk factors [17]. The underlying mechanism remains unclear but may be related to altered coagulation profiles in cirrhosis, which can confer a form of “natural anticoagulation,” reducing the incidence of coronary thrombosis [18]. Additionally, cirrhotic patients often have lower systemic vascular resistance and altered lipid metabolism, potentially contributing to a reduced risk of atherosclerosis and subsequent AMI [18]. 

A major strength of our study is the utilization of a large, nationally representative database, allowing for a robust comparison of the outcomes between patients with and without cirrhosis. The adjustment for multiple confounders, including comorbid conditions and hospital-level factors, enhances the validity of our findings. However, the study also has limitations. As a retrospective observational study using an administrative database, it is subject to potential biases related to coding accuracy and unmeasured confounders. The NRD lacks granular data on disease severity beyond ICD-10 coding, limiting our ability to stratify outcomes. Lastly, long-term outcomes beyond the initial hospitalization were not assessed, warranting further studies to evaluate longer-term mortality and morbidity in this high-risk cohort.

## Conclusions

In conclusion, patients with liver cirrhosis undergoing ICD placement face significantly higher risks of inpatient mortality, major bleeding, pericardial complications, and AKI. These findings highlight the need for careful risk assessment and multidisciplinary management to improve outcomes. While ICDs remain crucial for preventing sudden cardiac death, clinicians should weigh their benefits against the increased procedural risks in cirrhotic patients. The lower incidence of AMI in this population suggests unique cardiovascular pathophysiology that warrants further study. Future research should focus on refining risk prediction models and exploring alternative management strategies for high-risk patients.

## Appendices

Appendix A 

**Table 3:** Supplementary Table: ICD-10 codes used ICD-10: International Classification of Diseases, Tenth Revision; CVA: cerebrovascular accident: pRBC: packed red blood cell: CAD: coronary artery disease: STEMI: ST-elevation myocardial infarction: NSTEMI: Non-ST-elevation myocardial infarction Source: [8]

Diagnosis	ICD-10 code
Implantable cardioverter defibrillator	0JH60FZ, 0JH608Z, 0JH63FZ, 02HK4KZ, 02HK3KZ, 0JH638Z, 0JH837Z, 0JH609Z, 0JH639Z, 0JH809Z, 0JH839Z
Cirrhosis	K74, K74.3, K74.4, K74.5, K74.6, K70.3, K70.30, K70.31, K74.6, K74.60, K74.69
Ischemic CVA	I63.00, I63.01, I63.011, I63.012, I63.013, I63.013, I63.019, I63.02, I63.03, I63.031, I63.032, I63.033, I63.039, I63.09, I65.01, I65.02, I65.03, I65.09, I66.01
Gastrointestinal bleed	I85.01, I85.11, K20.81, K20.91, K21.01, K22.11, K22.6, K25.0, K25.2, K25.4, K25.6, K26.0, K26.2, K26.4, K26.6, K27.0, K27.2, K27.4, K27.6, K28.0, K28.2, K28.4, K28.6, K29.01, K29.21, K29.31, K29.41, K29.51, K29.61, K29.71, K29.81, K29.91, K31.811, K31.82, K50.011, K50.111, K50.811, K50.911, K51.011, K51.211, K51.311, K51.411, K51.511, K51.811, K51.911, K55.21, K57.01, K57.11, K57.13, K57.21, K57.31, K57.33, K57.41, K57.51, K57.53, K57.81, K57.91, K57.93, K62.5, K92.0, K92.1, K92.2
Intracranial hemorrhage	I60, I60.0, I60.00, I60.01, I60.02, I60.1, I60.10, I60.11, I60.12, I60.2, I60.3, I60.30, I60.31, I60.32, I60.4, I60.5, I60.50, I60.51, I60.52, I60.6, I60.7, I60.8, I60.9, I61, I61.0, I61.1, I61.2, I61.3, I61.4, I61.5, I61.6, I61.8, I61.9, I62, I62.0, I62.00, I62.0, I62.02, I62.03, I62.1, I62.9
Pulmonary hemorrhage	R04.2, R04.89, R04.9
Other bleed	D69.9, D68.3, D68.32, D68.31, R58
pRBC transfusion	30230N0, 30230N1, 30233N0, 30233N1, 30240N0, 30240N1, 30243N0, 30243N1, 30233P0, 30233P1, 30230P0, 30230P1, 30240P0, 30240P1, 30243P0, 30243P1
Pericardial complications	I31.2, I31.3, I31.4
Hypertension	I10, I15.0, I15.1, I15.2, I15.3, I15.9
Diabetes mellitus	E08.0, E08.00, E08.01, E08.1, E08.10, E08.11, E08.2, E08.21, E08.22, E08.29, E10.3, E10.31, E10.311, E11.0, E11.00, E11.01, E11.1, E11.10, E11.11, E11.2, E11.21, E11.22, E11.29, E13.0, E13.00, E13.01, E13.1, E13.10, E13.11, E13.2, E13.21, E13.22, E13.29
Smoking	F17, F17.2, F17.20, F17.200, F17.201, F17.203, F17.208, F17.209, F17.21, F17.210, F17.211, F17.213, F17.218, F17.219, F17.29, F17.290, F17.291, F17.293, F17.298, F17.299
Peripheral vascular disease	I70.0, I70.1, I70.2, I70.3, I70.4, I70.5, I70.6, I70.7, I70.8, I70.9, I73.8, I73.9, I79.8, E11.0, K55.1, K55.8, K55.9, Z9862
Chronic kidney disease	N18.1, N18.2, N18.3, N18.30, N18.31, N18.32, N18.4, N18.5, N18.9
Heart failure	I50.20, I50.21, I50.22, I50.23, I50.30, I50.31, I50.32, I50.33, I50.40, I50.41, I50.42, I50.43, I50.9, I50.2, I11.0, I13.0, I13.2, I09.81, I25.5, I42.0, I42.5, I42.6, I42.7, I42.8, I42.9, I97.13, I50.8, I50.81, I50.82, I50.83, I50.84, I50.89, I50.810, I50.811, I50.812, I50.813, I50.814
History of CAD	I25.2, Z98.61, Z95.5, Z95.1, T82855, T82855A, T82855D, T82855S
Atrial fibrillation	I48.0, I48.1, I48.11, I48.19, I48.20, I48.21, I48.91, I48.2, I84.9
Acute kidney injury	N17.0, N17.1, N17.2, N17.8, N17.9, N99.0
STEMI	I21.0, I21.01, I21.02, I21.09, I21.1, I21.11, I21.19, I21.2, I21.21, I21.29
NSTEMI	I21.4, I22.2

## References

[1] Al-Khatib SM, Hellkamp A, Bardy GH (2013). Survival of patients receiving a primary prevention implantable cardioverter-defibrillator in clinical practice vs clinical trials. JAMA.

[2] Liu YB, Chen MK (2022). Epidemiology of liver cirrhosis and associated complications: current knowledge and future directions. World J Gastroenterol.

[3] Huang DQ, Terrault NA, Tacke F, Gluud LL, Arrese M, Bugianesi E, Loomba R (2023). Global epidemiology of cirrhosis - aetiology, trends and predictions. Nat Rev Gastroenterol Hepatol.

[4] Chahal D, Liu H, Shamatutu C, Sidhu H, Lee SS, Marquez V (2021). Review article: comprehensive analysis of cirrhotic cardiomyopathy. Aliment Pharmacol Ther.

[5] Modi A, Vohra HA, Barlow CW (2010). Do patients with liver cirrhosis undergoing cardiac surgery have acceptable outcomes?. Interact Cardiovasc Thorac Surg.

[6] (2024). National Readmissions Database. https://hcup-us.ahrq.gov/nrdoverview.jsp.

[7] Mohyeldin M, Allu S, Schmidt P, Shrivastava S, Parikh H, Khaja M (2024). Socioeconomic and demographic determinants of readmission rates in congestive heart failure patients: insights from the nationwide readmissions database. Cureus.

[8] (2004). ICD-10: International Statistical Classification of Diseases and Related Health Problems: tenth revision, 2nd ed. https://iris.who.int/handle/10665/42980.

[9] Kujovich JL (2015). Coagulopathy in liver disease: a balancing act. Hematology Am Soc Hematol Educ Program.

[10] Kaltenbach MG, Mahmud N (2023). Assessing the risk of surgery in patients with cirrhosis. Hepatol Commun.

[11] Fede G, Privitera G, Tomaselli T, Spadaro L, Purrello F (2015). Cardiovascular dysfunction in patients with liver cirrhosis. Ann Gastroenterol.

[12] Lin CH, Hsu RB (2014). Cardiac surgery in patients with liver cirrhosis: risk factors for predicting mortality. World J Gastroenterol.

[13] Schrier RW, Arroyo V, Bernardi M, Epstein M, Henriksen JH, Rodés J (1988). Peripheral arterial vasodilation hypothesis: a proposal for the initiation of renal sodium and water retention in cirrhosis. Hepatology.

[14] Garcia-Tsao G, Parikh CR, Viola A (2008). Acute kidney injury in cirrhosis. Hepatology.

[15] Belcher JM, Garcia-Tsao G, Sanyal AJ (2013). Association of AKI with mortality and complications in hospitalized patients with cirrhosis. Hepatology.

[16] Nadim MK, Kellum JA, Forni L (2024). Acute kidney injury in patients with cirrhosis: Acute Disease Quality Initiative (ADQI) and International Club of Ascites (ICA) joint multidisciplinary consensus meeting. J Hepatol.

[17] Hillerson D, Ogunbayo GO, Salih M, Misumida N, Abdel-Latif A, Smyth SS, Messerli AW (2019). Outcomes and characteristics of myocardial infarction in patients with cirrhosis. J Invasive Cardiol.

[18] Wu VC, Chen SW, Chou AH (2020). Nationwide cohort study of outcomes of acute myocardial infarction in patients with liver cirrhosis: a nationwide cohort study. Medicine (Baltimore).

